# Multidimensional Analysis of Food Consumption Reveals a Unique Dietary Profile Associated with Overweight and Obesity in Adolescents

**DOI:** 10.3390/nu11081946

**Published:** 2019-08-19

**Authors:** Vanessa M.B. Andrade, Mônica L.P. de Santana, Kiyoshi F. Fukutani, Artur T.L. Queiroz, Maria B. Arriaga, Maria Ester P. Conceição-Machado, Rita de Cássia R. Silva, Bruno B. Andrade

**Affiliations:** 1 Programa de Pós-Graduação em Alimentos, Nutrição e Saúde, Escola de Nutrição, Universidade Federal da Bahia, Salvador 40110-150, Brazil; 2 Instituto Gonçalo Moniz, Fundação Oswaldo Cruz, Salvador 40296-710, Brazil; 3 Multinational Organization Network Sponsoring Translational and Epidemiological Research (MONSTER) Initiative, Fundação José Silveira, Salvador 45204-040, Brazil; 4 Curso de Medicina, Faculdade de Tecnologia e Ciências, Salvador 41741-590, Brazil; 5 Escola Bahiana de Medicina e Saúde Pública (EBMSP), Salvador 40290-000, Brazil; 6 Curso de Medicina, Universidade Salvador (UNIFACS), Laureate Universities, Salvador 41720-200, Brazil

**Keywords:** dietary intake, food group, adolescents, dietary patterns, anthropometric status, multidimensional statistical analysis

## Abstract

There is a significant increase in overweight and obesity in adolescents worldwide. Here, we performed a cross-sectional study to examine the potential association between food consumption profiles and overweight in a large number of adolescents from Brazil. Sampling by clusters and conglomerates was carried out in students of public schools in Salvador, Brazil, between June and December 2009 and 1496 adolescents were evaluated. Data on socio-epidemiological data, anthropometric status and food consumption were captured. Multivariate analyses, such as hierarchical clustering and correlation networks, were used to perform a detailed description of food consumption profiles. There were differences in age and anthropometric status related to sex. Four clusters of food groups were identified based on the intake profile in the study population. No disparities in food intake were observed in individuals stratified by sex or anthropometric status. Furthermore, network analysis revealed that overweight or obesity were hallmarked by a selectivity in the ingestion of food groups that resulted in the appearance of inverse correlations of consumption, which was not present in eutrophic adolescents. Thus, overweight and obesity are associated with preferential choices of ingestion of specific food groups, which result in the appearance of inverse correlations of consumption. Such knowledge may serve as basis for future targeted nutritional interventions in adolescents.

## 1. Introduction

Adolescence is a transition period between childhood and adulthood, in which social and psychological changes occur, influencing social behavior [1]. The intense transformations experienced in this age range increase the risk of developing adverse physical and psychosocial health problems. For example, during the teenage period, an individual is more likely to express higher concerns related to their physical appearance [1], often resulting in initiation of inadequate diets that can be deleterious to health.

During this period of life, several factors influence dietary habits, such as meals outside household, family environment, social interaction, media influence and cultural values. A better understanding of the influence of such factors on adolescent dietary habits and behavior is important to support measures focused on health improvements in this population [2]. Assessments of dietary intake are important because they identify the nutrient composition of the diet and estimate accordance with the reference values, allowing the nutritional diagnosis and subsequent specific intervention/therapeutic plans. Currently, most studies use for the evaluation of food consumption, the determination of dietary patterns, rather than the investigation of isolated nutrients or specific food groups [3]. This justifies the fact that the dietary pattern better reflects the complexity involved in nutrition, since they are defined as the quantities, proportions, variety or combinations of different nutriments and the frequency with in which they are habitually consumed by the individuals [4,5,6]. In fact, this approach allows for the evaluation of dietary implications with different food groups and correlates with clinical conditions more completely than analysis of isolated nutrients or specific food types [3].

Current dietary practices of adolescents have been characterized by increased intake of foods with high total and saturated fats, sugars and salt and low intake of fruits and vegetables [1]. Thus, the adoption of inappropriate dietary practices seems to be a determinant of the health and nutritional status. Such nutritional imbalance potentially contributes to weight gain and the development of other chronic diseases in adolescence with occurrence in adult life, including type-2 diabetes, cardiovascular diseases and several types of cancer. Changes in the dietary pattern in adolescence, with strategic substitution of food groups and focus on more balanced and healthy food, have great potential as a modifier of risk of these diseases and serve as a tool to promote health.

Overweight and obesity are considered as one of the main morbidities affecting individuals worldwide, in both developed underdeveloped countries, and are one of the major causes related to health problems [7]. The prevalence of overweight and obesity worldwide has been recently described in a review conducted between 2013 and 2014 [8]. This study has shown that approximately 110 million children and adolescents (ages 2 to 19 years) are overweight. In a more recent survey, the World Health Organization (WHO) reported a significant increase in prevalence of such conditions, with 340 million adolescents being overweight and/or obese. This represents a global prevalence of overweight of 19% among boys and 18% among girls [9]. Overweight in adolescence is therefore a global pandemic with an exponential increase in prevalence in recent years, which highlights the importance of dietary interventions for the reversal of this condition.

In the present study, we employ a novel comprehensive analytical approach using Big Data and Systems Biology tools to extensively describe the dietary patterns of 1496 adolescents from the public-school system in Northeast Brazil. In addition, we examined the potential association between food consumption profiles and overweight in the study population. 

## 2. Materials and Methods

### 2.1. Study Design and Participants

We performed a cross-sectional study in the city of Salvador, state of Bahia, the third largest city of Brazil, with adolescents from state public schools, as part of a larger project performed between June and December 2009 that assessed psychosocial factors as elements that affect health, nutrition and cognitive development [10]. Students with age between 11 and 17 years old, of both sexes who regularly attended the school and had parental/guardian approval were eligible to participate. Pregnant women, nursing mothers and students with physical problems that precluded anthropometric evaluation were not included in this investigation.

**Figure 1:**
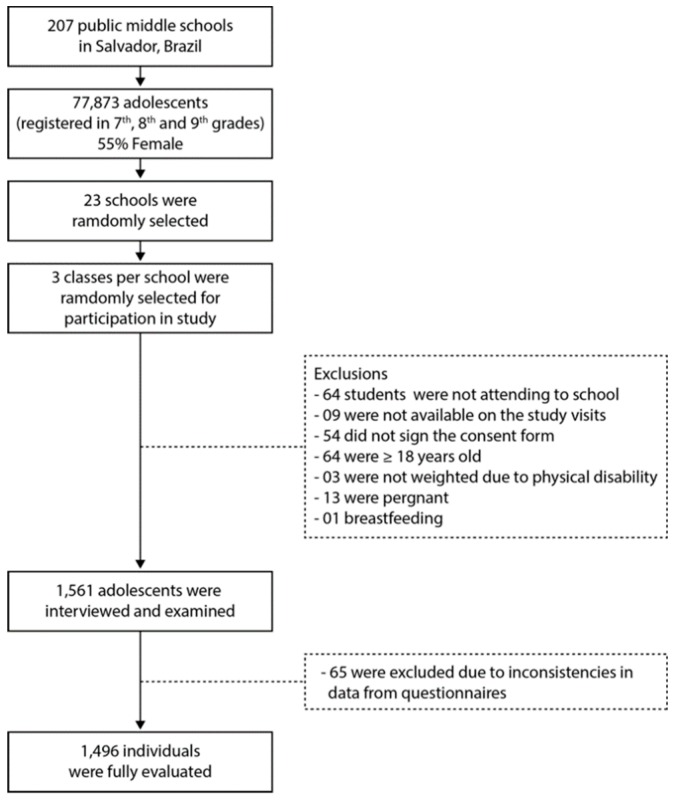
Study flowchart.

The sampling strategy was based on the simple random sampling technique, and the selection of the students was done by conglomerate in two stages: first, the selection of the schools, followed by the selection of the classes. The information from the public schools was provided by the state of Bahia Department of Education in 2007. Among the 207 state public schools in that year in Salvador, 23 were randomly selected and then three classes per school were chosen, in each class they were captured and interviewed an average of 30 students. Thus, 1561 students were evaluated and, after reviewing the questionnaires, 81 adolescents were not included in the study because they did not meet some inclusion criteria, namely: age greater than 18 years, presence of physical problem, gestation and lactation. Thus, the sample consisted of 1496 students. The delineation of study sample selection is shown in Figure 1.

Information on the economic conditions of families was provided by the parents. Stratification of the economic status followed the criteria specified by the Brazilian Federal Government (*Critério de Classificação Econômica*, Brazil) and predicted that a monthly average family wage below $75.00 US dollars is consider a poor economic status whereas a value above $145.00 denoted a relatively good economic status. Other data, such as age, sex, pubertal development following criteria published previously [11,12] and food consumption were self-reported by the students and recorded in appropriate standardized and previously validated questionnaires. Weight and height were measured. The data was captured in standardized report forms by a panel of trained nutrition technicians. 

### 2.2. Dietary Evaluation

The food intake was assessed using the semi-quantitative food frequency questionnaire (FFQ), consisting of 97 food items. The questionnaire used was constructed to fit the reality of the students of public schools in Salvador and later validated by our group [13]. The FFQ was applied directly to the students, who reported on their food consumption outside and inside the household. The frequencies of consumption of these food items were provided by adolescents and had the following options in response: Never/rare; 1 to 3 times a month; 1 time per week; 2 to 4 times a week; ≥4 times per week. In addition, the number of times a teenager consumed these food items was investigated. After the data collection, by using food composition and nutrition tables, we standardized the consumed quantities of each food and/or preparations referred to units of weight (g) and or volume (mL), which were used for calculation of the daily consumption of the food registered with the FFQ. Using this approach, data collected to measure consumption of food in a month (total month consumption) was deconvoluted to infer daily consumption (divided by number of days in a given month) as previously described [13]. Industrialized foods and/or preparations that were not included in the tables were searched via internet directly on the manufacturer’s website or through recipes. Thus, it was possible to obtain a proxy of the daily total food consumption in grams by calculations based on weekly and monthly consumption. For the statistical analysis of food consumption, the 97 food items that composed the FFQ were grouped according to the similarity in nutritional composition and food habits of the population of the Northeast of Brazil resulting in 14 previously defined food groups: Sugar and sweets, typical Brazilian dishes, sweetened beverages, fast food, oils, milk and dairy, meat, processed meat products, rice and cereals, roots, beans and legumes, vegetables, fruits and coffee. The approach used for creating these groups is described in Table 1.

### 2.3. Assessment of Anthropometric Status

Participants were weighed on a portable digital scale (Master Balancas, Goiania, Brazil), and their height was measured using a Leicester Height Measure portable stadiometer (Seca, Hamburg, Germany). The weight of the uniform (100 g) was subtracted during the analysis. WHO reference tables (2007) [14] with percentile values of the body mass index (BMI = weight [kg]/height [m^2^]) were used to assess the anthropometric status according to age and sex. In addition, the 2006 WHO criteria were used to categorize the anthropometric status: underweight (<3rd percentile), normal weight (≥3rd percentile and <85th percentile, the reference category), overweight (≥85th percentile and <97th percentile), or obese (≥97th percentile). 

### 2.4. Statistical Analysis

Descriptive statistics were performed to characterize the study population. Continuous variables were tested for Gaussian distribution using the D’Agostino-Pearson test. No variables exhibited normal distribution. Thus, medians and interquartile ranges (IQR) were used as measures of central tendency and dispersion. All comparisons were pre-specified. The non-parametric Mann-Whitney *U* test was used to compare distributions of continuous variables between two analytical groups whereas the Kruskal-Wallis test with Dunn’s multiple comparisons post-test was used to compare more than two groups. Categorical variables presented as percentage were compared using the Pearson’s chi-square test. Furthermore, we performed a number of additional analyses employed for Big Data and Systems Biology to provide novel insights in data visualization and interpretation. 

With the initial objective of evaluating the simultaneous consumption of food groups in the sample (“Which food groups presented similar consumption profile in the study population?”), a bi-directional, unsupervised, hierarchical cluster analysis was performed using Ward’s methodology (grouping both individuals and food consumption in grams) with bootstrap. For this analysis, a heat map was constructed with total consumption values in grams for each food group. Clustering was based on normalized consumption data in z-scores of the mean overall consumption of each food group. This analysis results in identification of clusters of individuals based on the similarity of consumption of the various food groups in grams. In this approach, dendrograms, which represent Euclidean distances and infer similarity [3]. A constellation plot was used to perform 2-dimension visualization of the clusters created by the Hierarchical analysis [15]. In this analysis, distance also infers similarity in overall consumption of food groups. 

Correlations between dietary intake values were assessed using the Spearman test. Spearman correlation matrices were constructed for each subgroup of individuals. The matrices were submitted to 100X bootstrap [16,17]. Bootstrapping was used to estimate more realistically the distribution of the correlations per group of individuals, accounting for multiple measures/comparisons using random sampling methods. This is a widely used approach employed in multidimensional analyses to increase accuracy of the statistical findings. Using this approach, only statistically significant correlations (*p* < 0.05), with values of Spearman rank (rho [r]) > ±0.5 (considered to be strong in the present study in pre-specified assumption) and that remained significant in at least 50% of the bootstraps were included in the network analyzes. The density of connections was calculated on each bootstrap and represents the following formula: *L*/(*N* × [*N*−1]/2), where *L* represents the number of statistically significant correlations (*p* < 0.05) and *N* is number of nodes (parameters). The network density, therefore, infers the number of significant correlations in relation to the total possible number of correlations in the matrix [16,17]. These values on network density were compared between groups using the Kruskal-Wallis test with Dunn’s multiple-comparison post-test. In the correlation network analyzes, the identification and characterization of nodes were performed comparing the number of correlations statistically significant for each food group in the different subgroups of individuals. Heat maps were constructed to optimize visualization of different patterns of food group consumption and nodal relationships in the networks.

Study power was calculated using JMP 13.0 (SAS, Cary, NC, USA). The predicted study total sample size per group to have a study power of 90% and alpha value of 5% and to find at least 1.5 fold-variation in overall consumption of at least 1 food group between the distinct anthropometric strata was 50, much lower that the total number of individuals recruited. Differences with *p* values lower than 5% after adjustment for multiple comparisons using the Holm-Bonferroni’s method considered statistically significant. Statistical analyzes were performed using GraphPad Prism 8.0 (GraphPad Software, La Jolla, CA, USA), JMP 13.0 and R 3.5.0 (R Foundation, Vienna, Austria). 

### 2.5. Ethics Statement

The research project was approved by the Ethics and Research Committee of the Institute of Collective Health of the Federal University of Bahia (protocol no. 002-08CEP/ISC). Written informed consent was obtained from all participants or their legally responsible guardians, and all clinical investigations were conducted according to the principles expressed in the Declaration of Helsinki. 

## 3. Results

### 3.1. Characteristics of Participants

The characteristics of the study participants are depicted in Table 2. Among the 1496 students enrolled, 642 were males (42.9%) and 854 females (57.1%). The median age was 14.3 years (interquartile interval: 13.1–15.5). Female participants were on average younger than male individuals (*p <* 0.0001; Table 2). Approximately half of the study population sample reported a poor family social economic status, with no difference between the subgroups stratified by sex (*p* = 0.3956). Regarding anthropometric status, the majority of adolescents was classified as eutrophic (*n* = 1155, 77.2%), whereas 8.8% (*n* = 132) were overweight and 5.9% (*n* = 89) were obese (Table 2). Female adolescents exhibited higher median BMI values than male participants (*p* = 0.006, Table 2). In addition, the overall frequency of the distinct anthropometric statuses was different between male and female participants (chi-square *p*-value: 0.005, Table 2). Hence, male individuals showed to be more frequently underweight than females (10.4% vs. 6.2%, respectively). With regards to pubertal development, the majority of the participants were in the post-pubertal stage (*n* = 1040, 69.5%) with a higher frequency of the most advanced stage of sexual maturation in females and pre-pubertal stage among boys (*p <* 0.001; Table 2).

### 3.2. Evaluation of Food Consumption Profiles

**Figure 2:**
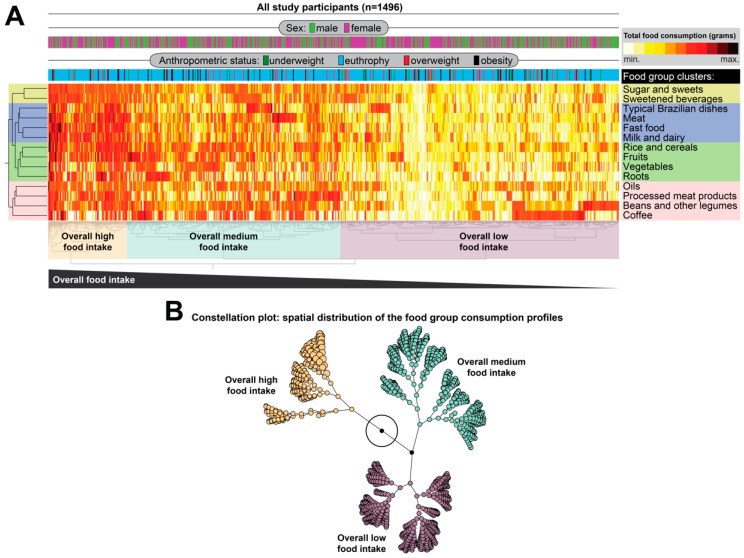
Analysis of the consumption of dietary groups using hierarchical cluster. The total consumption in grams obtained for each food group was calculated. (**A**) Two-way hierarchical cluster analysis (Ward’s method, unsupervised, with 100X bootstrap), in which the dendrogram represent Euclidean distance, was used as an approach to identify similarity profile of the consumption of distinct food groups. Using this approach, it was possible to identify four clusters of food groups that exhibited similar patterns of consumption in the general population. Three main subgroups of participants baes on overall food consumption was observed. (**B**) Constellation plot of the hierarchical clusters shows similarities between subgroups of study participants stratified by overall food intake.

The dietary items mentioned by the participants in their records were grouped into 14 food groups as described in Methods and depicted in Table 1. In order to initially understand the simultaneous consumption of different food groups, unsupervised cluster analysis was performed, in which the food groups were sorted according to similarity of food consumption in grams in the study participants (Figure 2A). Using this approach, it was possible to identify four clusters of food groups. Individuals who reported consuming more beverages also consumed more sugar and sweets (Figure 2A). Typical Brazilian dishes, meat, fast food and milk and dairy formed a large group of foods with a similar consumption profile. Rice and cereals, fruits, vegetables and roots formed the third group, and oils, processed meat products, beans and other legumes and coffee completed the fourth group of food consumption (Figure 2A). Of note, the overall profile of food consumption was not able to distinguish male from female study participants (Figure 2A), indicating that sex did not impact the total intake of the different food groups evaluated. The hierarchical cluster analysis was also used to simultaneously test whether individuals presenting with different anthropometric statuses would be grouped separately based on the overall food consumption profile. Interestingly, this statistical approach revealed that adolescents with divergent anthropometric statuses did not exhibit a distinct dietary profile when all the food groups were considered (Figure 2A). It was possible to detect three major groups of study participants based on the overall food consumption profile. In one smaller group (*n* = 300, which represented approximately 20% of the study participants), the overall consumption of the different food groups was high whereas two other groups displayed relative medium (*n* = 569, 38%) or low consumption (*n* = 627, 42%). Again, no preferential grouping related to sex or anthropometric status was observed between the three main clusters of food consumption (Figure 2A). A constellation plot, which is used to display 2-dimmension visualization of the clusters, demonstrated that the group of individuals who exhibited high consumption profile was more divergent than the other 2 groups of adolescents who had middle or low consumption (Figure 2B). 

**Figure 3:**
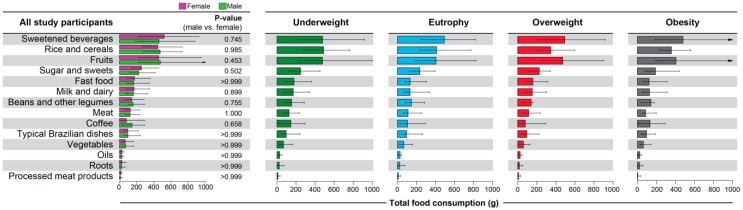
Consumption of food groups in individuals according to gender and anthropometric status. Data represent median and interquartile range of total consumption of each food group in grams. Left panel: For each food group, data were compared between males and females using the Mann-Whitney U test. Right panel: Distribution of the data in the groups of individuals with different anthropometric states.

Furthermore, we directly compared the individual food groups in the entire population and ranked based on total consumption. We found that sweetened beverages composed the most consumed food group in all the 1469 individuals, followed by rice and cereals, fruits, sugar and sweets and fast food (Figure 3). The least representative groups were vegetables, oils, roots and processed meat products. There were no differences in individual food group consumption between male and female participants (Figure 3, left panel). We further performed additional univariate analyses adjusted for multiple comparisons to try to delineate the factors which were associated with the different anthropometic statuses (Table 3). We found that underweight individuals presented more frequently with pre-pubertal or pubertal development, whereas post-pubertal adolescents were more common in the groups of overweight or obesity persons (chi square *p* < 0.001). Intriguingly, analyses revealed that total consumption of each food groups (measured in grams) was not different between the distinct groups of antropometric status (Figure 3, right panel and Table 3). 

### 3.3. Network Analyses of Food Consumption

Analyzes of dietary group intake had so far failed to reveal substantial, absolute quantitative differences between subgroups of adolescents stratified according to sex or anthropometric status. No single food group provides all the nutrients required for good health; thus, it is essential to eat a variety of foods with different vitamins and nutrients to fulfill all vitamins and nutrient necessities. A balanced consumption of the different food groups is described to be ideal to promote health. The next step was to examine the correlation profiles between the ingestion of the different food groups using network analysis [16,17]. This statistical approach makes possible to evaluate if augmented consumption of a given food group is followed by preferential increases or decreases in consumption of other groups, indicating dietary preferences of subgroups of individuals. We first tested direct correlation between the consumption of different food groups with BMI values and found no statistically significant relationship (Table 4), arguing that the consumption of a given individual food group was not associated with variation in BMI in the study population.

**Figure 4:**
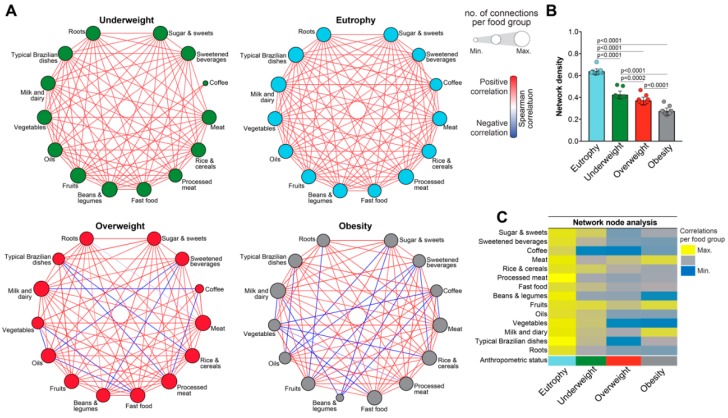
Network analysis of the correlations of consumption of food groups according to the anthropometric status. (**A**) Spearman correlation matrices were constructed for each subgroup of individuals stratified by anthropometric status. The matrices were submitted to 100X bootstrap. Only statistically significant correlations (*p* < 0.05 after adjustment for multiple comparisons), with rho (r) values >0.5 and that remained significant in at least 50% of the bootstraps were included in the network analyzes. (**B**) The network density, inferring significant number of correlations, was calculated on each bootstrap and the values were compared between the groups using the Kruskal-Wallis test with Dunn’s multiple comparisons post-test. The *p* values of the comparisons are shown. (**C**) Node analysis was performed comparing the number of correlations statistically significant for each food group in the different subgroups of individuals.

When the study participants were grouped according to the anthropometric status, it was observed that the great majority of the correlations was positive, indicating that the increased consumption of a given food group was related to greater the consumption of other groups (Figure 4A). Indeed, among eutrophic individuals, only positive correlations among consumption of foods were observed. A similar pattern of correlations was found in the group of underweight individuals (Figure 4A). Importantly, the number of negative correlations of the food groups was more expressive as the anthropometric status moved towards obesity. In addition, among individuals with thinness a lower number (*n* = 2) of correlations involving coffee intake was found (Figure 4A).

When examining the Overweight group, more peculiarities were found. A number of negative correlations were observed between ingestion of food groups (Figure 4A). Thus, the higher the intake of typical Brazilian dishes, the lower the intake of rice and cereals and processed meat. Increased intake of sweetened beverages was negatively associated with intake of oils as well as with beans and legumes. In addition, the lower intake of vegetables correlated with increased consumption of oils and fast food (Figure 4A). Among individuals with Obesity, once again several negative correlations between the consumption of several food groups were observed, demonstrating that specifically in this category, there is preferential intake of food groups in relation to others. For example, the consumption of vegetables was inversely correlated to the consumption of fast food, processed meat, rice and cereals, coffee and milk and dairy. Consumption of beans and legumes was also inversely proportional to the intake of milk and dairy products, sugar and sweets, sweetened beverages and coffee (Figure 4A).

Further analysis of the network densities in each of the 100 bootstraps performed in the correlation matrices built from the individuals with different anthropometric statuses revealed a reduction in the number of correlations between the consumption of the food groups in non-eutrophic conditions (Figure 4B). The lowest average network density was found among obese individuals (Figure 4B). These results indicate that there are quantitative (in numbers of statistically significant correlations) and qualitative (positive vs. negative correlations) changes in dietary profiles in conditions such as overweight and obesity. Finally, a node analysis was performed to identify which food groups had their consumption most related to the consumption of other foods. It was noted that among the eutrophic study participants, all food groups contributed in a similar way to a number of significant correlations. In underweight adolescents, the number of correlations changed and there was a slightly greater predominance of fruits, rice and cereals and sugar and sweets, and a great reduction of the importance of the consumption of coffee in the correlation matrices. In overweight participants, the consumption of meat and fruit formed the most relevant nodes. These groups were also among the most relevant in individuals with obesity, together with the consumption of milk and dairy (Figure 4C). Thus, the analysis of correlation networks was able to characterize quantitative and qualitative relations of consumption of the different food groups, highlighting differences related to the anthropometric status.

## 4. Discussion

In the present study, we performed novel multidimensional analyses adapted from the Big Data and Systems Biology fields to delineate the dietary patterns associated with overweight and obesity in a large number of adolescents from public school system in Brazil. The results presented here add to the current knowledge in the field as they highlight that adolescents with overweight or obesity, instead of exhibiting higher consumption of individual food groups, present an unbalanced dietary profile hallmarked by relative selective food consumption. 

The findings presented here revealed several differences male and female study participants. Female subjects were on average younger and had higher BMI values when compared to those of the opposite sex. Boys had a higher frequency of thinness/underweight. In addition, differences were observed in the referred stage of sexual maturation, with females presenting more frequently as post-pubertal, while males had a higher frequency of pre-pubertal. There was also no difference in level of economic condition between male and female participants. Thus, sexual maturation stage could have a higher influence on the anthropometric status than socioeconomic status. This influence was also observed in previous studies [18,19,20,21].

The first exploratory description of dietary intake has been performed here by hierarchical cluster analysis. The similarity profile for each food group was identified, based on food intake grams, and four food groups were identified. Thus, the amount of sweetened beverage ingested was similar to that of sugar and sweets (group 1). Other food groups that presented similarity in the amount of consumption were typical Brazilian dishes, meat, fast food and milk and dairy (group 2). Rice and cereals, fruits, vegetables and roots formed the third group of consumption, whereas oils, processed meat products, beans and legumes and coffee formed the fourth. These results suggest that the formation of food groups with similarity of consumption reflects a specific dietary profile. The formation of food habits is complex, and depends on associated factors such as biological, economic, food supply and availability [22]. The teenagers’ dietary habits are strongly determined by the environment in which they coexist, the influence of parents and friends, the food preparations, as well as their flavor. The taste of food is one of the factors that most interferes in the food choices of adolescents, that is, adolescents who have preference for foods with inadequate nutritional value may increase their consumption [22,23].

Although cluster analysis is a robust methodology for the identification of dietary patterns [24], few studies have used hierarchical grouping analysis and/or k-means for this purpose, mainly in adolescent populations [25,26,27,28]. Other studies evaluated eating patterns of adolescents but using factorial analysis [29,30,31,32]. In the area of nutritional epidemiology, there is a growing interest in innovative methods that bring more inferential information with advantages over conventional methods to better understand the complexity of eating practices. Traditionally, the evaluation of the food pattern occurs through factor analysis or principal components. This approach has the advantage of resulting in linear scores with adequate statistical power, however such scores are abstract and there is a limitation in understanding what they really mean [33]. Other methods include post-reduction regressions [34], Gaussian models [33] and, more recently, hierarchical grouping analysis [23].

The present study used clustering analysis because this method has the advantage of providing a clear description of exactly what groups of individuals are consuming [5,24,35,36] because the individual belongs to a single cluster, which is useful to help in the implementation of nutritional interventions. One of the few limitations of this method is the low power to detect associations with clinical outcomes when the sample size is small [3], however this is not the case of the present study. In addition, the FFQ is not 100% reliable as it depends on memory and it does not record consumption of food groups not listed in the questionnaire, leading to potential sub-notification of food intake. Regardless, the results presented here are robust because they result from a mixture of powerful statistical analyzes that, together, define the details of the food consumption of the adolescents related to the anthropometric status.

Although the present study detected differences in anthropometric profile and other characteristics between male and female participants, no differences were found in the total consumption of the different dietary groups. This means that, on average, boys and girls presented similar food group consumption. Additional analyzes of mean consumption by anthropometric failed to identify statistically significant differences. Thus, in the study participants, the average consumption of food groups does not seem to have been influenced by sex or anthropometric status. Additional studies using similar analyzes in populations of individuals with different age groups are needed to clarify whether the absence of dissimilarity in mean consumption among the various subgroups evaluated here is dependent on age range or another characteristic not examined in the present study.

The various complementary analyzes outlined a detailed food consumption profile, both from the point of view of the individual and the food groups in the study population. We used multidimensional statistical techniques to evaluate the balance of the intake of the different food groups in order to infer the quality of the diet. The Spearman correlation networks were able to define particularities regarding the relationships between differential food consumption. Similar approaches based on correlations have already been published in nutrition [37], however, not in the form of statistical interaction networks, which are more common in transcriptomic analyses [38] and immunology [16,17]. The present study is therefore innovative by using such techniques to visualize consumption profiles in pre-specified groups of individuals. These methodologies were able to highlight correlations that have not been identified with other statistical approaches. Regarding the anthropometric status, the network analysis revealed at least three main results: (i) A large number of statistically significant correlations were observed among the different food groups in all categories, indicating that the individual’s diet is a coordinated process that implies the simultaneous consumption of several food groups; (ii) in eutrophy and underweight, the great majority of the correlations were positive, indicating that the higher the consumption of a certain food group, the greater the consumption of other groups in general; (iii) in overweight and obesity, it was noticed the appearance of several negative correlations between the ingestion of food groups, demonstrating that there is preferential intake of some foods in relation to others. Intriguingly, in univariate analyses presented in Table 3 and Figure 3, individuals with different anthropometric status could not be distinguished based on total consumption of each individual food group when examined separately. In converse, among individuals with overweight or obesity, several negative correlations between the consumption of several food groups were observed in the network analysis. This finding indicates that rather been simplistically hallmarked by increased consumption in grams of one or more food groups, weight gain is associated with a preferential choice in the ingestion of food groups that is not present in eutrophy. Thus, although the average amount of food intake is not different in those who are overweight or obese compared to normal, dietary food selection potentially implies nutritional imbalance that results in weight gain. 

## 5. Conclusions

In summary, our study revealed that the presence of overweight or obesity is associated with the preferential choice of ingestion of specific food groups, resulting in the appearance of inverse correlations of consumption, which is not present in eutrophy and underweight. Such knowledge may serve as a basis for future investments in the field of nutritional epidemiology in Brazil.

## Figures and Tables

**Table 1:** Food groups according to similarity in nutritional composition, consumed by adolescents enrolled in the study.

Food or Food Groups	Food Items from the Food Frequency Questionnaire
1. Sugar and sweets	Sugar, chocolate powder, homemade sweets, industrialized sweets, stuffed biscuit, candies, chewing gum, lollipops, chocolate bar, gelatin, ice cream and popsicle (cream and/or chocolate).
2. Sweetened beverages	Normal, diet or light soda, artificial juice, carbonated drinks, artificial refreshment, energy drink and liquid or powdered sweetener.
3. Typical Brazilian dishes	Acarajé and abará ^a^, vatapá ^b^, caruru ^c^, feijoada ^d^, dobradinha ^e^, feijão tropeiro ^f^ and coconut milk.
4. Fast food	Fried potatoes, potato chips, pizza, lasagna, ketchup, ready-made soups, sandwich, industrialized salty snack, instant noodles, ready-to-eat sauce and pizza-ready sauce.
5. Oils	Butter, margarine, vegetable oil, mayonnaise, olive oil, palm oil.
6. Milk and dairy	Whole milk powder or liquid, skimmed milk powder or liquid, fermented milk, yogurt (whole, diet or light), chocolate ready, yellow cheese, white cheese, cream cheese, creamy curd (whole or light).
7. Meat	Bovine (fried or cooked), chicken with or without skin (fried or cooked), cooked or fried fish, seafood, viscera, chicken egg (fried or cooked), dehydrated meat (Jerky beef).
8. Processed meat products	Ham, mortadella, sausage, calabrese.
9. Rice and cereals	Bread (white or whole), rice (white or whole), noodles (white or whole), cassava flour, farinaceous (oats, wheat germ), milk or nest meal, green corn or couscous of corn, popcorn salted), homemade cake, box cake, granola, biscuit (salted or sweet), pasta soup.
10. Roots	Cassava, sweet potato, potato.
11. Beans and legumes	Beans, peanuts, nuts and walnuts.
12. Vegetables	Lettuce, cabbage, cabbage, pumpkin, carrot, tomato, chayote, gherkin, beet, okra, vegetable salad.
13. Fruits	Pineapple, avocado, acerola, silver banana, ground banana, cashew, jackfruit, papaya, mango, apple, watermelon, melon, orange, tangerine, strawberry, fruit juice or fruit pulp, acai-berry.
14. Coffee	Coffee and tea.

^a^ Dishes made of beans and shrimp, deep-fried in palm oil, the only difference is that abará is steamed, while acarajé is fried. ^b^ Creamy paste prepared with bread, shrimp, coconut milk, finely ground peanuts and palm oil. ^c^ Made from okra, onion, shrimp, palm oil and toasted nuts (peanut and/or cashew). ^d^ Stew of beans with beef and pork. ^e^ Dish made from a cow’s flat white stomach lining. ^f^ Cattleman’s Beans.

**Table 2:** Distribution of the demographic, economic, biological, and behavioral characteristics total and according to the sex of the adolescents enrolled in the study.

Characteristic	Total (1496) *n* (%)	Male (642) *n* (%)	Female (854) *n* (%)	*p*-Value
Age–years (median and IQR) *	14.3 (13.2–15.5)	15.6 (13.4–15.8)	14.2 (13.2–15.2)	<0.001
**Socioeconomic status**				0.634
Good economic condition	727	319 (51.5)	408 (49.2)	
Poor economic condition	721	300 (48.5)	421 (50.8)	
**BMI–Kg/m^2^** (median and IQR) *	18.9 (17.2–21.0)	18.7 (16.9–20.7)	19.1 (17.4–21.2)	0.006
**Anthropometric status**				0.005
Underweight	120	67 (10.4)	53 (6.2)	
Eutrophy	1155	471 (73.4)	684 (80.1)	
Overweight	132	59 (9.2)	73 (8.6)	
Obesity	89	45 (7.0)	44 (5.1)	
**Pubertal development**				<0.001
Pre-pubertal	126	122 (19.0)	4 (0.5)	
Pubertal	325	162 (25.2)	163 (19.1)	
Post-pubertal	1040	354 (55.1)	686 (80.3)	

BMI: body mass index; IQR: interquartile range. * Difference of values between male and female were compared using the Mann-Whitney *U* test. Qualitative variables were represented by frequency and compared using the Pearson’s chi-square test. Missing data: Socioeconomic status = 48.

**Table 3:** Factors associated with the distinct anthropometric status.

Parameter	Total *	Underweight	Eutrophy	Overweight	Obesity	Adjusted *p*-Value **
	*n* = 1491	*n* = 119	*n* = 1151	*n* = 132	*n* = 89	
**Age (years)**	14.3 (13.2–15.5)	14.3 (13.4–15.2)	14.4 (13.3–15.5)	14.0 (12.8–15.4)	14.0 (13.2–14.9)	0.31
**Sex**						
Female	851 (57.1)	52 (43.7)	682 (59.3)	73 (55.3)	44 (49.4)	1.00
Male	640 (42.9)	67 (56.3)	469 (40.7)	59 (44.7)	45 (50.6)	
**Socioeconomic status**						1.66
Good economic condition	724 (50.2)	54 (47)	549 (49.3)	67 (51.9)	54 (62.8)	
Poor economic condition	719 (49.8)	61 (53)	564 (50.7)	62 (48.1)	32 (37.2)	
**Pubertal development**						<0.01
Pre-pubertal	125 (8.4)	21 (17.6)	86 (7.5)	10 (7.6)	8 (9)	
Pubertal	325 (21.9)	47 (39.5)	237 (20.7)	25 (19.1)	16 (18)	
Post-pubertal	1036 (69.7)	51 (42.9)	824 (71.8)	96 (73.3)	65 (73)	
**Food or food group consumption (grams)**						
Sugar and sweets	243.3 (130.2–436.1)	235.2 (125.5–398.4)	247.9 (136.9–452.9)	227.2 (110.5–341.7)	190.5 (81.7–432.3)	0.31
Sweetened beverages	480.4 (200.0–915.3)	493.3 (240–820)	480.4 (200–920)	493.3 (213.3–920.1)	480 (187.3–973.4)	1.00
Typical Brazilian dishes	97.3 (49.3–239.8)	95.9 (48–262.6)	98.6 (50.6–240.9)	95.9 (49.3–223.8)	95.9 (39.6–189.4)	1.00
Fast food	170.4 (80.3–352.7)	134.3 (86–303.3)	180 (81.6–363.5)	158.8 (80.2–310.3)	123.3 (66–307.3)	0.42
Oils	29.3 (11.5–51.1)	28.7 (9.3–44.5)	29.7 (12.3–51.9)	26.6 (9.2–44.7)	24.7 (9.3–44)	0.73
Milk and dairy	166.4 (70.9–337.6)	132.8 (65.6–338.4)	171.9 (72.2–344.3)	154.3 (74.2–296.4)	126.9 (57–304.4)	0.99
Meat	122.7 (64.0–236.7)	112 (60–253.3)	127.3 (65.3–236.7)	115.3 (56–234)	86.7 (54.7–192)	0.98
Processed meat products	11.0 (5.5–33.0)	11 (5.5–33)	11 (5.5–33)	11 (2.7–30.2)	5.5 (2.7–33)	0.31
Rice and cereals	460.7 (261.8–730.6)	412.7 (235.2–772.8)	490.6 (280.9–765.4)	437.4 (193.2–598.8)	487.4 (244.4–531.6)	0.08
Roots	24.7 (6.8–71.8)	24.5 (6.8–77.9)	27 (6.8–74.4)	17.7 (6.8–49.1)	24.5 (6.8–56.4)	1.00
Beans and legumes	148.8 (78.0–286.0)	148.8 (83.8–286)	154.6 (78–286)	153 (52–158.9)	143 (47.8–177.8)	0.09
Vegetables	67.3 (23.1–161.2)	66.1 (26.6–159.7)	69.2 (23.2–165.5)	60.8 (11.6–127.3)	64.2 (29.6–137.5)	0.98
Fruits	465.7 (218.3–988.4)	405.3 (189.9–825.6)	479.1 (224.3–999.8)	472.3 (248.2–894.8)	407 (195.6–1111)	1.00
Coffee	106.7 (13.3–293.3)	106.7 (26.7–293.3)	146.7 (13.3–293.3)	80 (13.3–293.3)	133.3 (13.3–293.3)	1.00

* From 1496 adolescents, only 1491 had all data available to analyze the food group. ** *p*-values were adjusted for multiple measurements using the Holm-Bonferroni method. Differences of values in continuous variables (represented by median and interquartile range [IQR]) between food groups were compared using the Kruskal-Wallis test with Dunn’s multiple comparisons post-test. Qualitative variables were represented by frequency and compared using the Pearson’s chi-square test.

**Table 4:** Spearman correlation analysis between total consumption of each food group and BMI values.

Food Group	All Individuals		Underweight		Eutrophy		Overweight		Obesity	
	rho Value	*p* Value	rho Value	*p* Value	rho Value	*p* Value	rho Value	*p* Value	rho Value	*p* Value
Sugar and sweets	−0.03	0.25	0.02	0.81	0.02	0.52	0.00	1.00	0.12	0.28
Sweetened beverages	0.03	0.28	0.06	0.48	0.03	0.29	−0.08	0.37	0.02	0.88
Typical Brazilian dishes	0.01	0.65	0.03	0.75	0.03	0.34	−0.03	0.72	0.11	0.31
Fast food	0.01	0.84	0.00	0.99	0.01	0.79	0.01	0.91	0.11	0.30
Oils	0.05	0.09	−0.02	0.82	−0.06	0.06	0.02	0.84	0.01	0.89
Milk and dairy	−0.03	0.31	0.01	0.88	−0.03	0.38	−0.05	0.57	0.01	0.94
Meat	−0.01	0.84	−0.04	0.69	−0.01	0.78	0.07	0.41	0.02	0.89
Processed meat products	0.07	0.08	0.00	0.97	0.07	0.06	−0.01	0.92	0.16	0.14
Rice and cereals	0.08	0.05	0.06	0.52	−0.05	0.12	−0.09	0.31	0.07	0.50
Roots	0.02	0.41	0.11	0.22	−0.01	0.62	−0.07	0.41	0.14	0.19
Beans and legumes	−0.02	0.49	0.10	0.27	0.00	0.88	0.03	0.72	0.14	0.20
Vegetables	−0.04	0.13	0.03	0.73	−0.05	0.08	−0.04	0.67	0.10	0.38
Fruits	0.01	0.70	0.06	0.49	0.02	0.55	−0.12	0.19	0.00	0.97
Coffee	−0.04	0.17	−0.07	0.43	−0.03	0.39	−0.08	0.38	0.06	0.57

Data represent Spearman rho as well as *p*-values of each correlation in each indicated study group.
